# vis–NIR and XRF Data Fusion and Feature Selection to Estimate Potentially Toxic Elements in Soil

**DOI:** 10.3390/s21072386

**Published:** 2021-03-30

**Authors:** Asa Gholizadeh, João A. Coblinski, Mohammadmehdi Saberioon, Eyal Ben-Dor, Ondřej Drábek, José A. M. Demattê, Luboš Borůvka, Karel Němeček, Sabine Chabrillat, Julie Dajčl

**Affiliations:** 1 Department of Soil Science and Soil Protection, Faculty of Agrobiology, Food and Natural Resources, Czech University of Life Sciences Prague, Kamycka 129, Suchdol, 16500 Prague, Czech Republic; coblinskijoao@gmail.com (J.A.C.); drabek@af.czu.cz (O.D.); boruvka@af.czu.cz (L.B.); nemecekk@af.czu.cz (K.N.); jerabkovaj@af.czu.cz (J.D.); 2 Helmholtz Centre Potsdam, GFZ German Research Centre for Geosciences, Telegrafenberg, 14473 Potsdam, Germany; saberioon@gfz-potsdam.de (M.S.); chabri@gfz-potsdam.de (S.C.); 3 Remote Sensing Laboratory, Department of Geography and Human Environment, Porter School of Environment and Earth Science, Tel Aviv University, Tel Aviv 69978, Israel; bendor@tauex.tau.ac.il; 4 Department of Soil Science, Luiz de Queiroz College of Agriculture, University of Sao Paulo, Padua Dias Avenue, 11, CP 9, Piracicaba 13418-900, Brazil; jamdemat@usp.br

**Keywords:** soil contamination, vis–NIR spectroscopy, XRF spectroscopy, data fusion, feature selection, univariate filter, genetic algorithm

## Abstract

Soil contamination by potentially toxic elements (PTEs) is intensifying under increasing industrialization. Thus, the ability to efficiently delineate contaminated sites is crucial. Visible–near infrared (vis–NIR: 350–2500 nm) and X-ray fluorescence (XRF: 0.02–41.08 keV) spectroscopic techniques have attracted tremendous attention for the assessment of PTEs. Recently, the application of fused vis–NIR and XRF spectroscopy, which is based on the complementary effect of data fusion, is also increasing. Moreover, different data manipulation methods, including feature selection approaches, affect the prediction performance. This study investigated the feasibility of using single and fused vis–NIR and XRF spectra while exploring feature selection algorithms for the assessment of key soil PTEs. The soil samples were collected from one of the most heavily polluted areas of the Czech Republic and scanned using laboratory vis–NIR and XRF spectrometers. Univariate filter (UF) and genetic algorithm (GA) were used to select the bands of greater importance for the PTE prediction. Support vector machine (SVM) was then used to train the models using the full-range and feature-selected spectra of single sensors and their fusion. It was found that XRF spectra alone (primarily GA-selected) performed better than single vis–NIR and fused spectral data for predictions of PTEs. Moreover, the prediction models that were derived from the fused data set (particularly the GA-selected) enhanced the models’ accuracies as compared with the single vis–NIR spectra. In general, the results suggest that the GA-selected spectra obtained from the single XRF spectrometer (for As and Pb) and from the fusion of vis–NIR and XRF (for Pb) are promising for accurate quantitative estimation detection of the mentioned PTEs.

## 1. Introduction

The soil science society of America (SSSA) defines soil contaminant as any substance in soil that exceeds naturally-occurring levels and poses human health risks. Indeed, soil contamination refers to a process, in which non-pedogenic components with no relation to the soil’s natural formation accumulate in soil and cause adverse effects on plant growth as well as animal and human health [1]. Soil contamination has been increasing as a result of anthropogenic influences, such as urbanization, industrialization, population, and agricultural growth [2]. As a major group of soil contaminants, potentially toxic elements (PTEs) (e.g., As, Pb, Cd, and Cr ) can be primarily found in soluble and adsorbed fractions [3]. These elements can present risks to animal and human health by entering the food chain, water, soil, and plants [4,5]. Their persistent nature and long biological half-lives disturb the soil balance and threaten the health of animals and plants, which reduces the seed quality and root growth of some species [6,7]. Hence, assessing PTE concentrations in soil is of particular interest for their effective monitoring and further remediation.

PTEs are conventionally measured using laboratory chemical methods, such as soil extraction or digestion, followed by atomic absorption spectrometry (AAS) or inductively coupled plasma (ICP) analysis, which are time-consuming and expensive procedures and not always suitable for a large number of soil samples. However, proximal spectroscopic techniques, such as visible–near infrared (vis–NIR: 350–2500 nm) and X-ray fluorescence (XRF: 0.02–41.08 keV), increase the proficiency of soil data collection and they deliver more information on soil variation in less time and at a lower cost compared with conventional methods [8,9]. vis–NIR spectroscopy is a passive technique that reflects diffuse electromagnetic radiation at the surface of the sample, measured as reflectance, and the absorbed energy causes the vibration of the molecular bonds in the target material (in this case, soil); as a result, the energy of the reflected signal is lower than that of the originally received signal [10]. The proportion of the incident radiation that is reflected by the target material is sensed through vis–NIR spectroscopy, and the amount of light absorbed can be evaluated, which enables the assessment of soil attributes [11]. It acquires one spectrum per second that includes useful information for several soil attributes extraction [12]. XRF spectroscopy is an active technique that is based on the excitation of inner electrons, which causes the emittance of radiation (fluorescence) from the target material (in this case, soil). The fluorescence is detected by an XRF spectrometer as a signal, and elements are measured on the basis of the type and strength of this signal [9,13].

Over the last two decades, vis–NIR [6,14,15] and, recently, XRF [9,16] spectroscopy have been individually used for the detection of PTEs in soil. However, a single soil sensor does not provide a fully comprehensive characterization of soil PTEs. Moreover, according to [17], soil assessment with a single sensor is sometimes less stable because of the complex nature of soil, so it is reasonable to search for other techniques that are complementary and appropriate for concurrent analyses [18]. Recently, the fusion of spectral data derived from both sensors (vis–NIR + XRF) has been suggested to more efficiently provide soil attribute information [19] within a greater spectral range [13]. The fusion of these technologies has already been demonstrated to be capable of predicting certain soil attributes, such as carbon and nitrogen [20,21], salinity [22], and petroleum hydrocarbons [23]. However, only a few recent studies have focused on the potential of fused data from these two sensors and compared their performance with independent data obtained from the individual sensors for soil PTE estimation [13,24].

The application of fused vis–NIR and XRF proximal techniques has recently increased, because of progress in technology, computational power, spectral pre-processing methods, fusion approaches, machine learning algorithms, and the selection of bands of greater importance for the prediction of PTEs. Different data manipulation techniques, including the direct fusion of the data from single sensors [25], the direct fusion of selected spectral features [26], the fusion of models developed from individual sensors [27], and the outer product analysis (OPA) method [24] have been used to integrate different spectroscopic techniques and they have shown a wide range of prediction performances. In fact, the choice of an appropriate method and its ability to handle data are essential in obtaining a robust, reliable, and accurate prediction [28,29]. Accordingly, comparing the estimation ability of single sensor data sets (vis–NIR and XRF) and the fused sensor data (vis–NIR + XRF) through the application of their full-range and feature-selected spectra would be highly useful for improving soil PTE prediction. Hence, further works on finding more effective manipulation methods of the spectral data are needed for better exploitation of the techniques.

Over the last few decades, soils in several regions of the Czech Republic, as well as in other countries, have been heavily affected by industrial activities. Therefore, this study aimed to test the capability of individual vis–NIR and XRF spectra as compared with the fused data set to detect and determine six PTE concentrations (As, Cd, Cu, Pb, Zn, and Mn) in the soils of one of the most heavily polluted areas of the Czech Republic. The specific objectives were: (i) to investigate the feasibility of the full-range spectra and the selected features from the individual spectrometers (vis–NIR and XRF) for prediction of the PTEs, (ii) to examine the PTEs prediction accuracies using fusion of the instruments’ full-range spectra (vis–NIR + XRF) and their spectra derived from the selected features of each spectrometer (vis–NIR + XRF feature-selected), and (iii) to compare the performance of the single vs. fused spectra while exploring the potential of univariate filter (UF) and the genetic algorithm (GA) for feature selection and support vector machine (SVM) regression for model calibration. Because the spectral range of each technique offers specific contributions to the prediction procedure, it is expected that the complementary effect of data fusion will have a promising influence on the PTE models’ predictive performance. Moreover, the improvement of PTE quantification is anticipated because bands of greater importance for prediction are selected using feature selection techniques.

## 2. Materials and Methods

### 2.1. Study Area, Soil Sampling, and Soil Analysis

**Figure 1:**
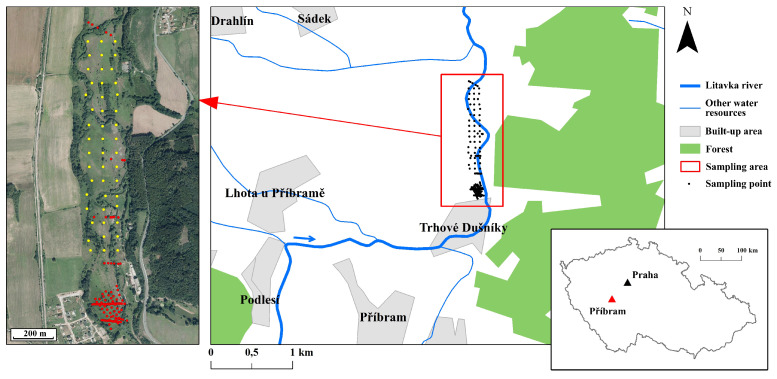
The Czech Republic and location of Příbram in the country, the location of the sampling area, and the sampling points. Sampling points’ colors are distinguishing between different sampling strategies (at different periods); yellow points are the new sampling points and red points represent previously analyzed, but newly collected and re-analyzed, sampling points.

Soil samples from the Příbram district (49${}^{\circ }$71${}^{\prime }$ N; 14${}^{\circ }$01${}^{\prime }$ E) in the Czech Republic were analyzed to achieve the study goals. Příbram is a town in central Bohemia that is located 50 km to the south of Prague (Figure 1). As a result of long-term ore mining and metallurgical activities, the area of the Litavka river alluvium is heavily polluted, with PTEs from three sources: (i) naturally increased metal content because of the specific composition of parent rocks, (ii) atmospheric deposition from a smelter, and (iii) floods of water polluted with metal-processing wastes [30,31]. According to the World reference base of soil resources [32], soils of the area are predominantly characterized as Fluvisols, Gleysols, and, in areas that are slightly elevated relative to the alluvium, Cambisols.

One-hundred fifty-eight soil samples (Figure 1) were collected in April 2018 using both grid and transect sampling designs with manual augers. The selected sample size had sufficient coverage of the predictor space, and the samples were suitable indicators for the area to which the models were applied. The position of each sampling point was recorded by a GeoXM (Trimble Inc., Sunnyvale, CA, USA) receiver with an accuracy of 1 m. For each sample, about 2 kg soil was taken at depths 0–20 cm as a composite sample over an area of 5×5 m and then air-dried, ground, sieved (⩽2 mm), and thoroughly mixed before analysis. The pseudo-total contents of six key PTEs, namely, As, Cd, Cu, Pb, Zn, and Mn, were extracted using aqua regia (ISO, 11466:1995). The solution was 10-fold diluted with deionized water (conductivity 18.2 MΩ) and then filtered through 0.45 μm Nylon Membrane Disc Filters (Thermo Fisher Scientific, Waltham, MA, USA) prior to analysis. The extract was analyzed for PTE content by ICP-OES iCAP 7000 (Thermo Fisher Scientific, Waltham, MA, USA). The concentration measurements were subjected to quality control (QC) using the SRM 2711 (Montana II soil) internal reference material (National Institute of Standards and Technology, Gaithersburg, MD, USA). The obtained values were in good agreement with the reference data; the recovery differences were generally less than 10% (*n* = 3). Using the same method as in the evaluation of PTEs, total iron (Fe), and using the rapid dichromate oxidation technique [33], soil organic carbon (SOC), were also determined as auxiliary data for further use (correlation with PTEs), since they are well-known spectrally active soil properties [5,34] and they strongly adsorb PTEs [35]. The samples and standards were matrix-matched to compensate for matrix effects that influence analytical response [36]. All of the analyses were performed in triplicate.

### 2.2. Spectral Data Acquisition and Pre-Processing

#### 2.2.1. vis–NIR Spectroscopy

**Figure 2:**
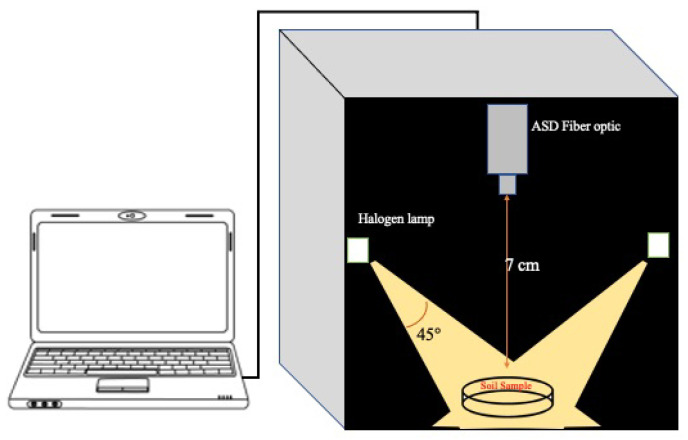
vis–NIR spectra measurement setup.

The vis–NIR (350–2500 nm) spectroscopic measurements were acquired using an ASD FieldSpec 4 Pro FR (ASD Inc., Denver, CO, USA) in an harmonized dark box environment with fixed illumination and geometry (Figure 2) that was developed at the German Research Center for Geosciences (GFZ) [12]. The spectral resolution of the spectroradiometer was 3 nm for the range of 400–1050 nm and 10 nm for the region 1050–2500 nm. The radiometer bandwidth from 350 nm to 1000 nm was 1.4 nm, while it was 2 nm for 1000–2500 nm, and the data were interpolated every 1 nm over the whole wavelength range.

The instrument was run for 60 min to warm up the spectrometer and lamps. The spectral measurements were acquired with an 8${}^{\circ }$ fore-optic and ∼7 cm height nadir viewing sensor above the target, producing a ∼1 cm ground instantaneous field of view (GIFOV) in the middle of the sample. The samples were placed in 5 cm diameter Petri dishes to form 2 cm layers of soil and they were scanned in the center (three replications each, rotating and steering the sample, flattening before each measurement). The spectrometer was optimized using a Spectralon™ white reference (Labsphere, North Sutton, NH, USA) at the beginning and end of each batch of five soil sample measurements. In order to minimize systematic errors during measurements, the internal soil standard (ISS) sample that was introduced by [37] was scanned after the Spectralon™ white reference at the beginning of each batch and also before the next Spectralon™ measurement at the end of each batch.

For spectral data pre-processing, noisy portions between 350 nm and 400 nm, as well as 2451 nm and 2500 nm, were removed, leaving spectra in the range from 400 nm to 2450 nm (2051 variables) for further processing. The resulting spectra were then smoothed using the Savitzky-Golay approach [38] with a second-order polynomial fit and window size of 11 wavelengths in order to remove the artificial noise within the working spectral range [6,39], and the first derivative was generated and used to remove baseline offset and enhance spectral features [40]. In this study, influential outliers in a set of predictor variables were detected by applying ensemble sparse partial least squares [41].

#### 2.2.2. XRF Spectroscopy

**Figure 3:**
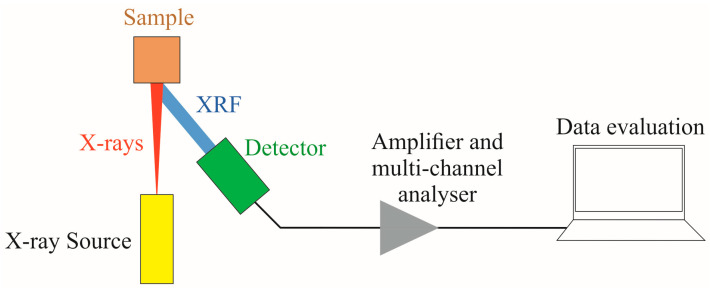
X-ray fluorescence (XRF) spectra measurement setup.

An Olympus Delta Premium XRF (Olympus, Center Valley, PA, USA) spectrometer was used to collect XRF spectra in the range of 0.02–41.08 keV (Figure 3). The measurements were acquired in Soil Mode, which emits three beams of 50 keV, 40 keV, and 15 keV per scan [27]. The XRF spectrometer was calibrated by the manufacturer sets for soil. The soil samples were placed in 10 mL plastic cups, covered with 4 μm thick polypropylene film, and then set on the XRF laboratory stand [13]. Afterward, the soil surface was directly scanned by the instrument in triplicate with a scanning time of 60 s per scan (amounting to 180 s total time), and the average results were used for the direct PTE concentration and raw XRF spectra.

A recent study was conducted on the same soil samples and focused on the determination of the As concentration, which was directly derived from the XRF sensor and then compared with the result obtained using the ICP-OES conventional laboratory technique [42]. The study confirmed that XRF was capable of predicting the As concentration in soil at comparable levels of accuracy to the ICP-OES method. In the current study, the XRF spectra were employed for further analysis. To pre-process the extracted raw spectra, the range of the spectra was first reduced to 0.64–14.99 keV (716 variables) to exclude the low-energy bands, followed by the application of the Savitzky-Golay spectral smoothing algorithm with a second-order polynomial fit and window size of 11 wavelengths and the first derivative to improve the modelling efficiency [24].

### 2.3. Feature Selection

A practical approach for improving the models’ robustness and accuracy is the elimination of irrelevant variables and redundancies in the data and the selection of relevant spectral features [26]. According to [43], feature selection reduces spectral complexity and maintains only useful wavelengths that are highly correlated with the predicted variables. In this study, the UF [44] and the GA [45] approaches were used to select the most relevant vis–NIR and XRF spectral features for PTE assessment. These selected features, instead of the full-range spectra, were then used to develop models to predict the concentrations of PTEs from each sensor.

The UF technique selects those variables that have the greatest correlation with the response, thereby only observing the properties that are inherent to the data without using any clustering algorithms to guide the search for relevant features. The relevance score for the feature is then calculated individually and it does not include feature interactions. The main advantages of this method over other feature selection approaches are the processing speed and the ability to process large data sets [46]. The GA is a popular heuristic optimization algorithm, as introduced as an evolutionary algorithm [47], which uses a flexible search strategy to randomly select an initial set of spectral variables and optimize this set by analyzing multiple combinations of features and their interactions [1]. In other words, the GA aims to reach the global optimum for a problem by retaining the best individuals in the population using mutation and crossover operations. The GA procedure can be summarized into five steps: (i) the coding of all variables, (ii) initiation of the population, (iii) evaluation of the responses, (iv) reproduction, and (v) mutations [48]. An initial set of spectral variables is typically randomly selected, and this set is then optimized by evaluating many combinations of spectral features by following the principles of reproduction and mutation [49]. More details on the GA approach can be found in [50]. The parameters that were adopted in this study were primarily related to the population size of each generation (5), the number of iterations (50), deletion group (5), crossover probability (0.8), and mutation probabilities (0.1). The Caret package of the R software (R Development Core Team, Vienna, Austria) was used to implement feature selection [51].

### 2.4. Data Fusion

For the fusion of the data sets that were obtained from the vis–NIR and XRF spectroscopy techniques, three types of data integration were considered: (i) full-range spectra from single sensors (vis–NIR + XRF full-range) [25,52], which was termed as low-level fusion by [24], (ii) spectral features selected from each sensor by UF (vis–NIR + XRF UF-selected), and (iii) spectral features that were selected from each sensor by GA (vis–NIR + XRF GA-selected). The fusion of selected features from the two sensors was termed middle-level fusion by [24]. For each approach, the data sets were directly concatenated in a single table as a variable matrix and defined as the fused sensor data set [53].

### 2.5. Model Construction and Evaluation

Each data set was randomly divided into training (75%) and testing (25%) data sets [12]. The training data set was used to develop the regression model, and the testing data set was used to validate the developed model’s generalization capability [54]. The spectral modelling of the selected soil components was performed using SVM. The implementation of the applied method for spectroscopic modelling has previously been explained in detail by the authors [55,56]. A basic grid search approach was applied to tune SVM’s hyperparameters (i.e., Cost function and Sigma). All of the spectroscopic models were validated using 10-repeated 10-fold cross-validation [57].

The final accuracy prediction was evaluated using standard model evaluation metrics: the coefficient of determination (R${}^{2}$), root mean squared error (RMSE), and mean error (ME) or bias. R${}^{2}$ is the proportion of variation in the response that is explained by the regression model, and RMSE describes the model’s prediction capability. The bias or ME represents the error of means and is independent [58,59].

## 3. Results

### 3.1. Descriptive Statistics, Spectral Response, and Correlation of PTEs, Total Fe, and SOC

Table 1 presents the number of samples after outlier detection and the results of descriptive statistical analysis of the measured pseudo-total content of PTEs, total Fe, and SOC content, including the mean, median, minimum (Min), maximum (Max), standard deviation (SD), coefficient of variation (CV), and skewness.

The samples that were used in this study contained wide ranges of all elements (Table 1). According to the pollution levels for the Czech soils reported by [60,61,62,63], the soils of the area are classified as highly polluted, with mean values of 200 mg/kg, 24.2 mg/kg, 54.6 mg/kg, 1803 mg/kg, 2217 mg/kg, and 2380 mg/kg for As, Cd, Cu, Pb, Zn, and Mn, respectively (Table 1). The data distribution features were illustrated by the SD values, where all SDs were lower than the corresponding mean values for all examined PTEs. The lowest PTE CV, which is the degree of variability in the element’s concentration in the soil, was obtained for Cu (CV = 35%) because of its moderate variability and greater homogeneity [64]. However, the CVs of other PTEs were ≥ 43%, indicating that their values were moderately to highly variable in this study area [64,65]. Additionally, the skewness was used to test the normality of the PTE data and revealed that most of the elements were approximately normally distributed, with skewness values that were close to 0 (Table 1). The Fe statistics had a high mean (20,973 mg/kg), relatively low CV (26%), and skewness of 0.40. The soil samples indicated the average SOC contents of 3.1% ± 1.1% (SD) with a distribution that shows rather low variability (CV = 35%).

**Figure 4:**
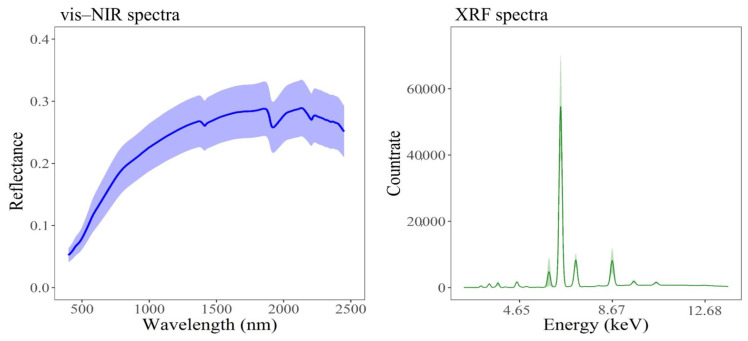
Representative soil mean spectra (bold lines) and their variance (shaded areas) of vis–NIR and XRF.

Figure 4 highlights the representative mean soil spectra with their variance acquired from both spectrometers. Both spectra have the typical shape and pattern of the vis–NIR and XRF spectra of soil samples. In the vis–NIR spectrum, a gradual increase over the range of 400–700 nm and an almost flat segment between 700 nm to 1000 nm can be observed, which are characteristic patterns of SOC and Fe-oxide mixtures. A few of the observed absorption features can also be attributed to the presence of water (at 1400 nm and 1900 nm) and clay minerals (at 2200 nm) [34]. In the XRF spectrum, the count rates represent the emitted spectrum intensity at each photon energy, so they are the basis for quantitative analysis as well as the built-in algorithm [66]. Some visible peaks can be seen in the spectrum that reflect the signals for Mn and heavy elements of Zr and Sr, although the highest count rate in the XRF spectrum is at around 6–7 keV (Figure 4), which is the signal for Fe [21].

**Figure 5:**
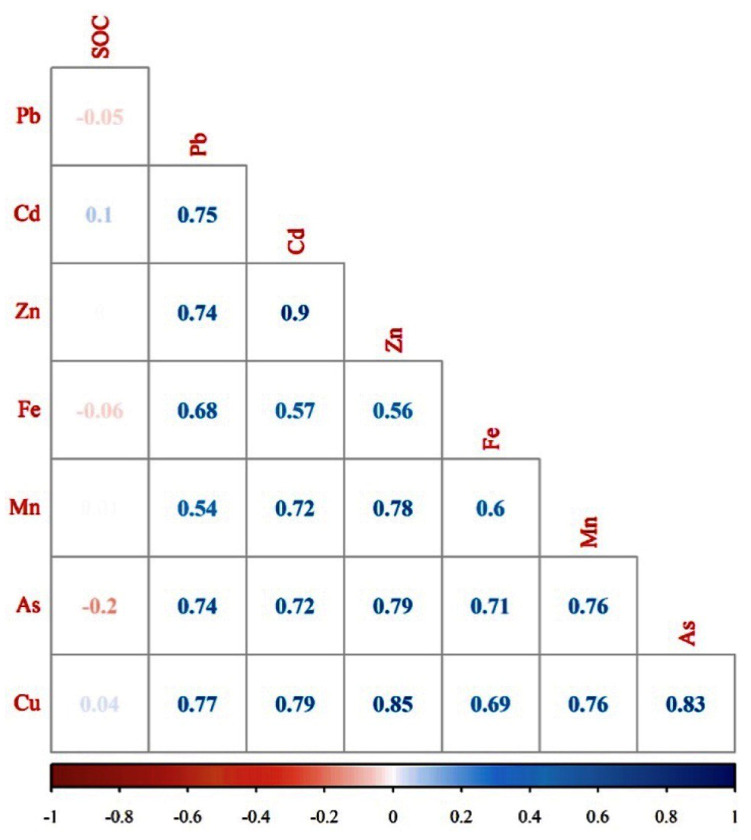
The Pearson correlation coefficients between soil PTEs, total Fe, and SOC.

The Pearson correlation coefficients (*r*) between the examined PTE concentrations and between the PTEs, total Fe, and SOC contents of the soil samples were calculated (Figure 5). All of the examined soil PTEs were positively correlated with each other, implying their robust mutual dependence [12], with the highest Pearson correlation coefficient between Cd and Zn (*r* = 0.90). Generally, the highest correlation among contaminants was observed between Zn and other PTEs (0.55 ⩽*r*⩽ 0.90). Fe, as a spectrally active element and as a secondary product related to the impact of PTEs on the soil, was significantly and positively correlated with all PTEs, with *r* values between 0.55 and 0.70, as shown in Figure 5. Nevertheless, SOC demonstrated no (with Zn) or a very low correlation with all other elements, with the highest negative correlation with As (*r* = −0.20).

### 3.2. Estimation of PTEs Using the Single Spectrometers Full-Range Spectra

Table 2 highlights the results of the PTE prediction models using the non-linear SVM algorithm applied to the full-range spectra of individual sensors (vis–NIR and XRF).

It was found that the models used for the prediction of PTE concentrations by the vis–NIR full-range spectra were poor (R${}^{2}$ < 0.50) for Cd, Zn, and Mn, according to [67]’s classification. Other elements, namely As, Cu, and Pb, were better predicted (0.53 ≤ R${}^{2}$ ≤ 0.61), although they still cannot be fairly recommended for qualitative analysis. The estimation results obtained from the XRF full-range spectra were more satisfactory and considerably better (0.71 ≤ R${}^{2}$ ≤ 0.89) than those from the vis–NIR spectra, in which the prediction accuracies for Pb, Zn, and Mn were characterized as good [67]. The results shown in Table 2 also indicate that, when compared with other PTEs, soil Pb was the most accurately predicted element with confidence in both vis–NIR (R${}^{2}$ = 0.61, RMSE = 665 mg/kg, and bias = 64.3 mg/kg) and XRF (R${}^{2}$ = 0.89, RMSE = 382 mg/kg, and bias = −8.93 mg/kg) full-range spectra.

### 3.3. Estimation of PTEs Using the Single Spectrometers Feature-Selected Spectra

The entire vis–NIR and XRF pre-processed spectra that were used for predictions included 2051 and 716 spectral variables, respectively. In order to reduce multicollinearity and noise, the spectral dimension reduction was conducted by selecting the variables of greater importance. After feature selection using the UF and GA methods, fewer spectral variables of the vis–NIR and XRF spectra, as compared with their full-range spectra, were selected to model the PTEs. Table 3 shows the number of features selected by the UF and GA techniques for each data set.

Table 2 presents the results of the PTE estimation models that were developed using SVM coupled with the UF or GA feature selection method. These results show that the prediction models obtained using selected features were better for the XRF spectra (0.70 ≤ R${}^{2}$ ≤ 0.89) than vis–NIR (0.22 ≤ R${}^{2}$ ≤ 0.68), which is a similar trend to the results that were obtained from the full-range spectra. Table 2 also indicates that the estimations by the GA feature selection technique were more effective than those obtained using the UF for all elements when vis–NIR spectra were applied, and for As, Pb, Zn, and Mn, when XRF spectra were employed.

More importantly, using the feature-selected vis–NIR spectra improved the performance of the PTE estimation (except Cd), as compared with the vis–NIR full-range spectra (Table 2). A similar effect was observed in the prediction of As and Cu for XRF, when the GA was applied and in prediction of Cd and Cu, when UF was used. Although the increase in prediction accuracy using both techniques on the XRF spectra was not very noticeable.

### 3.4. Estimation of PTEs Using vis–NIR and XRF Data Fusion

The data fusion approach was employed and tested for developing the spectral models of the selected soil PTEs in this study. This involved concatenation of the vis–NIR and XRF full-range, UF-selected, and GA-selected spectra, followed by SVM modelling of the fused data. Table 4 presents the modelling results.

It can be seen that the fused spectra selected by the GA feature selection technique generally provided higher R${}^{2}$ and lower RMSE and bias than either the full-range or the UF-selected fused spectra (Table 4). This difference was more obvious in the estimation accuracy of Pb, although it was less remarkable in other PTEs.

The fusion of the vis–NIR and XRF spectra improved the prediction of PTE concentration (Table 4) as compared with the vis–NIR spectra alone, with and without the feature selection approaches (Table 2). In other words, models that were based on the fused spectra produced better predictions of more PTEs, when compared with those based on only the vis–NIR spectra. For instance, Cd that was poorly predicted using the single vis–NIR full-range or feature-selected spectra (0.22 ≤ R${}^{2}$ ≤ 0.25, 8.41 mg/kg ≤ RMSE ≤ 8.96 mg/kg, and −1.90 mg/kg ≤ bias ≤ 1.73 mg/kg; Table 2), was reasonably predicted by the vis–NIR and XRF fused spectra, with R${}^{2}$, RMSE, and bias values of 0.75, 4.04 mg/kg, and 0.44 mg/kg, respectively (Table 4). In contrast, as indicated in Table 2, the standalone XRF spectra (both full-range and feature-selected) led to the better performance of almost all PTE prediction models than the vis–NIR + XRF spectra, regardless of whether the full-range or feature-selected spectra were used (Table 4).

### 3.5. Comparison of Models Derived from Different Spectral Data Sets

**Figure 6:**
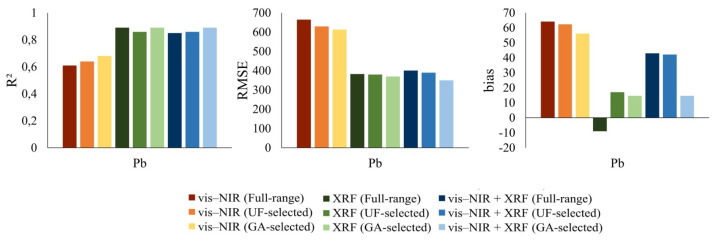
Pb (mg/kg) prediction model performance using different spectral data sets.

From the estimations using the spectrometers’ individual and fused full-range and feature-selected spectra (Table 2 and Table 4), the optimal models provided the best predictions for the Pb content of soil as compared with the other PTEs. In addition, among different PTEs, Pb is identified as a major soil pollutant [2]. Hence, this element was considered in the subsequent investigation to visualize the impact of the methods and input data sets on the prediction performance (Figure 6).

**Figure 7:**
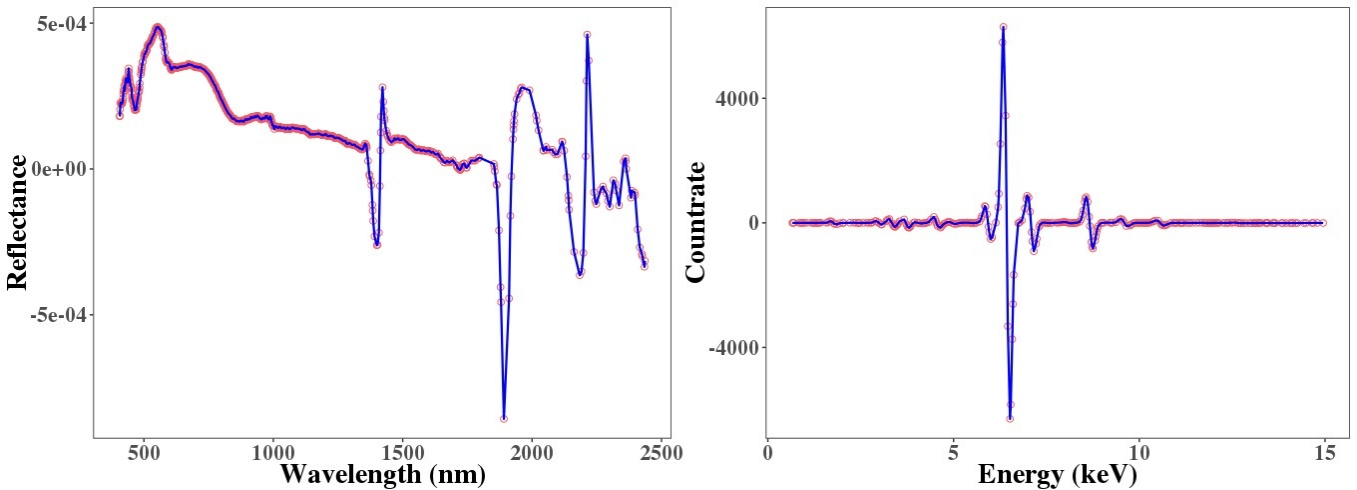
Feature selection by genetic algorithm (GA) from vis–NIR and XRF spectra (blue lines are the first derivative spectra, and the red dots are the selected features).

Figure 6 shows that the SVM models for Pb estimation provided different prediction accuracies, when using different sensors’ data sets. Pb was predicted from the single vis–NIR sensor full-range spectra with R${}^{2}$ of 0.61, RMSE of 665 mg/kg, and bias of 64.3 mg/kg. After feature selection, the redundant and irrelevant variables were discarded. Figure 7 clearly shows that GA was the more successful feature selection approach (than UF) for the reduction of variables (for Pb). It can be seen that, in vis–NIR spectra, the more useful features were in the ranges of 500–1300 nm and 1500–1700 nm. For XRF, it can be seen that the GA excluded most of the features between 5.22 KeV and 7.74 KeV as less important variables.

Therefore, when compared with the use of the single vis–NIR full-range spectra, the feature-selected spectra of single vis–NIR, particularly the GA-selected spectra, produced the better estimation of Pb (R${}^{2}$ = 0.68, RMSE = 613 mg/kg, and bias = 56.2 mg/kg). The XRF GA-selected spectra provided similar R${}^{2}$, but slightly lower RMSE values as compared with the XRF full-range spectra. Furthermore, the full-range and feature-selected spectra from XRF alone both provided better Pb prediction models than the single vis–NIR data. Spectroscopic full-range data fusion, as well as the fusion of the useful variables obtained from the spectra of two sensors, noticeably enhanced the prediction ability of the Pb models (0.85 ≤ R${}^{2}$ ≤ 0.89, 350 mg/kg ≤ RMSE ≤ 401 mg/kg, and 14.6 mg/kg ≤ bias ≤ 43.0 mg/kg) in comparison with the single vis–NIR data, while no substantial improvement was evident, when compared with the XRF standalone data sets. Generally, the optimal models with higher estimation accuracies and/or lower error were obtained using the single XRF (both full-range and feature-selected) as well as the fused GA-feature-selected spectra.

## 4. Discussion

The soils of Příbram area in the Czech Republic were characterized as strongly polluted by high accumulations of PTEs (As, Cd, Cu, Pb, Zn, and Mn) based on the pollution levels reported for Czech soils [61,62]. The total Fe content in the soils of the area was also high, ranging from 9670 mg/kg to 35,428 mg/kg. The soil samples indicated medium average SOC contents of 3.1% (Table 1). Hence, the intense peaks in the spectra of both spectrometers at specific wavelengths or energy levels (Figure 4) are not directly linked to the presence of PTEs. It has been proven that PTEs mostly do not have direct and recognizable spectral features within the vis–NIR region, and they may be indirectly detected via inter-correlation with the soil attributes that are spectrally active in this region and through their bonding with clay, SOC, and Fe, which are acquirable [14,34]. Thus, the reflectance spectra can be utilized for the indirect assessment of PTEs in soil samples via the spectrally active soil attributes and contaminant concentrations correlation. For example, reference [68] indicated negative correlation between Cr, Cu, Zn, and As and the absorption features of SOC, clay, and Fe-oxides. They also displayed correlations between Cd, Pb, and Hg with the spectral region that is related to SOC. Therefore, they mentioned that, by using soil proxy methods with reflectance spectroscopy, various soil PTEs can be monitored efficiently. In this study, Figure 5 confirmed the effect of Fe, which highlights the significant positive correlation of total Fe with all PTEs, indicating that they were closely bond to Fe [14]. This shows that Fe, as a spectrally active soil property, more significantly influenced the estimation of PTE concentrations from spectra and had higher priority to interact with the examined PTEs than SOC. These results were similar to the results of [2,12]. The reason for a more significant role of Fe as compared to SOC is perhaps because the content of inorganic components in dry soils is larger than the organic components and, hence, they can influence spectra more considerably, according to [69]. Nevertheless, reference [70] mentioned that Fe in mineral soils acts as a stable background for spectral response of SOC and, therefore, in these soils what can be seen as the spectral features of Fe are practically the spectral response of SOC and organic matter. Thus, the role of SOC cannot be ignored in the spectral modelling procedure of the examined PTEs in alluvial soils of the Czech Republic.

Soil PTE sensing with single vis–NIR full-range spectra has been widely explored with different ranges of modelling performances [6,12,14,54,71]. This study found poor accuracy (Table 2), particularly for Cd and Zn, when using vis–NIR spectroscopy alone. But As, Cu, and Pb were better (although still not very reasonable) predicted (0.50 < R${}^{2}$ < 0.65). According to previous studies that analyzed these elements (As, Pb, and Cu) using vis–NIR [1,12,24], successful modelling can be related to their strong correlations with other soil properties, including Fe (Figure 5), which has a spectral response in this range [11,34,72]. Regarding the poor statistics of Cd and Zn, reference [73] explained that a larger fraction of Cd and Zn penetrates the soil and precipitates from the solution phase of the spill. Hence, a great proportion of these elements is distributed in the soil profile by an independent process that prevents their detection by vis–NIR spectroscopy applied to topsoil samples. The estimation accuracy and error (R${}^{2}$, RMSE, and bias) for the prediction of PTEs using the single XRF data were better than those of the single vis–NIR data (Table 2); these findings are in agreement with the studies by [13,24,26]. In recent years, the accuracy of XRF devices has increased significantly, with limits of detection that are low enough to measure PTEs, according to [74]. Generally, they have a relatively wide dynamic range and are able to detect PTEs at any concentration, which is probably a reason for the successful XRF outputs. Moreover, XRF is based on electrons emitted from atoms, so it can be element specific. On the other hand, vis–NIR is based on molecular vibrations, not on specific elements and, thus, can predict the PTE content only indirectly through, for example, the soil components to which the PTEs are bound (e.g., SOC and Fe-oxides). Therefore, XRF is anticipated to provide better results. Despite the better results that were obtained from the single XRF spectra in this study, the remarkable advantages of vis–NIR over XRF spectroscopy in soil monitoring and assessment cannot be neglected. vis–NIR spectroscopy provides more soil attribute information from the same spectrum in one measurement [9]. In addition, while vis–NIR sensors allow for the acquisition of one spectrum per second, XRF devices are typically employed with time ranges between 60 s to 90 s. Furthermore, vis–NIR spectroscopy is more conveniently adopted by remote sensing [12]. These aspects support the potential use of vis–NIR spectroscopy as a very efficient technique in the prediction of soil properties, including soil PTEs.

The fused full-range spectrum (vis–NIR + XRF) was used to develop PTE prediction models in order to further explore the potential of fusing the data of both sensors to predict PTE concentrations (Table 4). The fused full-range data outperformed the prediction models derived from the single vis–NIR method in terms of accuracy and error. This can be linked to the ability of XRF to complement the information obtained from vis–NIR spectroscopy, as XRF spectra allow for a broad characterization of the soil constitution with a low detection limit [74]. However, data fusion was not found to be as successful as the standalone XRF outputs for most of the elements. This result can be associated with the poor results of the single vis–NIR data, which negatively influence the performance of the fused spectra, introducing a form of additional noise and, thus, reducing the accuracy compared with the single XRF data. Therefore, given a choice between using the full-range vis–NIR and/or XRF spectra, the use of XRF individually or in combination with vis–NIR can effectively predict soil PTEs, which is in correspondence with the findings of [19]. Some studies that integrated vis–NIR and XRF data sets found that the fusion was successful and it led to better model performances than either individual sensors for soil assessment, including the prediction of petroleum contamination [23] and PTEs [13,26], highlighting the feasibility of using both of them together. According to [13,53], the fusion of vis–NIR and XRF spectra, although resulting in a much larger data set that requires more computation time and memory than individual data sets, appears to be highly suitable, because more comprehensive information is available. Nevertheless, in the studies conducted by [24,27], no significant improvements were observed in the full-range spectra fusion (low-level fusion) results, when compared with those obtained using the individual sensors, particularly for As and Cd [24].

In the subsequent investigation, by employing the UF or GA feature selection algorithms, we tested the benefits and usefulness of spectral dimension reduction and the selection of an optimal set of features (instead of using the full-range spectrum) on PTE prediction models. After feature selection, the unimportant and less relevant bands or variables were discarded, the dimension of the spectra was reduced, and fewer variables of the vis–NIR and XRF spectra were selected (Table 3). These methods are generally useful, as their application can mitigate the computing issue and avoid potential over-fitting and the need for large calculation and memory capabilities for soil data analysis [47]. In this study, the model predictions had increased estimation accuracies and decreased errors, when using the feature selection approach on the single vis–NIR spectra (except for Cd) and when using the GA approach on the individual XRF full-range data set for monitoring As and Cu (Table 2). This can be linked to the ability of feature selection to reduce spectral complexity and only retain effective, highly correlated variables [43]. This is an important indicator of the efficiency of using feature selection techniques to simplify models with parsimonious data sets without a loss of prediction accuracy. Although the feature selection procedure requires additional time, this may be offset by the more accurate and stable predictions of PTEs that are produced by the applied models. The positive impact of feature selection on prediction performance was less pronounced for the fused spectra (vis–NIR + XRF), when compared with the sensors’ individual spectra (particularly single vis–NIR). It should be mentioned that, in the current study, the applied feature selection methods generally did not meet our expectations, since we expected more obvious improvement in prediction performances of all PTEs using either single or fused spectra. However, this was only partially achieved. According to [75], although variable selection may provide more reliable models, the reverse is also possible, since the selection of irrelevant variables can have a negative effect. Hence, studying more efficient feature selection methods, which can detect the most relevant wavelengths, can provide promising results.

In this study, the GA selected less spectral variables (Table 3) and it provided better results than UF (Table 2 and Table 4). The reason for the superiority of GA over UF is that, in contrast to univariate approaches, such as UF, the GA gains deeper insight into the spectral predictive mechanisms and the relevance of the spectral predictor variables for a successful calibration model [47]. In a study conducted by [76], GA and uninformative variable elimination (UVE) were applied to vis–NIR spectra to assess soil carbon. It was confirmed that using GA-selected variables instead of the full-range spectrum increased the accuracy of the models. The GA allows for an efficient search in high-dimensional and complex response surfaces, as stated by [77]. As an evolutionary algorithm, it has high potential to remove non-informative variables, determines the most relevant spectral discriminants, and creates a smaller data set in terms of the number of original spectral variables included and, thus, decreases the problem of multicollinearity and noise embedded in the spectra [47,49].

Overall, this study revealed that the single XRF data provided reliable results with R${}^{2}$ ≥ 0.80 for the assessment of Pb, Zn, and Mn. Although the standalone GA-selected spectra as well as the fused spectra (vis–NIR + XRF) coupled with the GA feature selection approach were the superior techniques for detecting As and Pb contamination in a Czech Republic’s case study with alluvial soils in an accurate and environmental-friendly manner. Moreover, when we used vis–NIR spectroscopy alone, employing the feature selection approaches (particularly GA) resulted in improved prediction performances (except for Cd). The prediction results of PTEs in this study indicated that the fusion of vis–NIR and XRF spectra provided comprehensive information, particularly when compared to vis–NIR single spectra. However, selecting featured variables and eliminating irrelevant features using GA and UF were less advantageous than they were expected to be and need more studies to be conducted testing more feature selection algorithms. This will bring an advancement in soil testing that can provide a considerable amount of additional soil information and it can render the implementation of soil monitoring schemes more feasible. This will also provide an opportunity to perform novel and accurate retrieval algorithms in operational processing chains for the global quantitative determination of soil contamination.

It should be stated that, in this study, processed dry samples were used for spectral measurements and model development, and the resulting models might not be suitable for vis–NIR and XRF measurements in the field, because the techniques are sensitive to soil surface disturbance factors, such as moisture and particle size distribution [16,78]. Therefore, it is necessary to assess the potential for using sensors fusion in-situ. Moreover, the increasing number of proximal and remote soil sensing instruments across the electromagnetic spectrum extends the possibility of using sensors fusion. Thus, future studies are required to focus on the fusion of these techniques as complementary data to assess multiple soil attributes for agricultural and environmental applications simultaneously. Finding solutions to avoid the classic disadvantage of data fusion related to handling large volumes of data from multiple sources should also be among the objectives of future investigations. Additionally, different machine learning algorithms, deep learning, and other fusion and feature selection approaches need to be investigated.

## 5. Conclusions

This study employed the fusion of vis–NIR and XRF soil scanning technologies and feature selection algorithms (UF and GA) to predict key PTEs (As, Cd, Cu, Pb, Zn, and Mn) in the soils of one of the most heavily polluted areas of the Czech Republic.

The results showed that: (i) when using the full-range data of individual sensors, XRF predicted all PTEs with an R${}^{2}$ larger than 0.71, which is better than the results that were obtained from vis–NIR; (ii) the predictions obtained from the sensors’ fused data set enhanced the models’ accuracies, when compared with the use of solely vis–NIR. Nevertheless, the single XRF data set provided better results than the fused spectra in the majority of the examined PTEs; (iii) the use of the GA method improved the estimation accuracies of As and Pb models as compared with the full-range spectra using either single or fused spectra; (iv) the escalating impact of feature selection on prediction performance was more pronounced for the individual vis–NIR spectra as compared with the XRF and fused spectra; and (v) Pb was the most accurately predicted element using all of the examined data sets with confidence in the individual and fused vis–NIR and XRF spectra.

The general conclusion of this study is that the GA-selected spectra of XRF alone and vis–NIR + XRF were the more efficient methods for assessing soil As and Pb of the study site. In this study, a better performance for single vis–NIR spectra was also observed when coupled with the spectral bands of greater importance, although the results were not yet favorable. There are some advantages of vis–NIR over XRF spectroscopy that cannot be neglected, including a shorter scanning time, the delivery of more soil attributes information from the same spectrum, and the convenience with which it is adopted by remote sensing. Therefore, when using solely vis–NIR spectroscopy for the prediction of PTEs, which is very common, employing feature selection approaches (e.g., UF and GA) is highly recommended.

## Figures and Tables

**Table 1:** Statistical description of measured pseudo-total content of potentially toxic elements (PTEs), total Fe, and soil organic carbon (SOC).

Element	No.	Unit	Mean	Median	Min	Max	SD	CV%	Skewness
As	150		200	197	4.50	492	102	51	0.3
Cd	152		24.2	24.5	1.60	48.4	10.3	43	0.0
Cu	151		54.6	53.7	13.2	104	19.6	35	0.3
Pb	152	(mg/kg)	1803	1656	37.9	4170	920	51	0.5
Zn	154		2217	2124	49.4	5351	1128	51	0.4
Mn	142		2380	2324	499	5720	1140	48	0.8
Fe	152		20,973	20,503	9670	35,428	5522	26	0.4
SOC	147	(%)	3.1	2.9	0.9	6.2	1.1	35	0.5

**Table 2:** Statistics of the prediction model performance for soil PTEs concentration (mg/kg) using the single spectrometers’ full-range and feature-selected spectra (validation data set).

Element	Sensor	Data Set	R${}^{2}$	RMSE	Bias
As	vis–NIR	Full-range	0.59	76.7	11.9
		UF-selected	0.55	78.5	13.8
		GA-selected	0.61	76.5	9.43
	XRF	Full-range	0.77	58.2	14.4
		UF-selected	0.70	66.4	15.8
		GA-selected	0.82	52.5	14.4
Cd	vis–NIR	Full-range	0.25	8.42	−1.90
		UF-selected	0.22	8.96	1.86
		GA-selected	0.25	8.41	1.73
	XRF	Full-range	0.73	4.98	0.38
		UF-selected	0.78	4.51	0.50
		GA-selected	0.74	5.05	0.08
Cu	vis–NIR	Full-range	0.53	13.9	2.30
		UF-selected	0.56	13.25	1.82
		GA-selected	0.58	13.2	1.73
	XRF	Full-range	0.71	10.8	−0.19
		UF-selected	0.78	9.91	−0.98
		GA-selected	0.76	10.10	0.06
Pb	vis–NIR	Full-range	0.61	665	64.3
		UF-selected	0.64	630	62.4
		GA-selected	0.68	613	56.2
	XRF	Full-range	0.89	382	−8.93
		UF-selected	0.86	379	17.1
		GA-selected	0.89	370	14.7
Zn	vis–NIR	Full-range	0.37	907	141
		UF-selected	0.37	939	205
		GA-selected	0.52	808	131
	XRF	Full-range	0.81	501	50.0
		UF-selected	0.79	520	58.8
		GA-selected	0.80	509	43.8
Mn	vis–NIR	Full-range	0.45	844	68.3
		UF-selected	0.47	835	67.8
		GA-selected	0.53	829	50.0
	XRF	Full-range	0.82	488	−21.3
		UF-selected	0.80	517	53.8
		GA-selected	0.83	509	−11.0

**Table 3:** Number of spectral variables selected by Univariate filter (UF) and genetic algorithm (GA) from vis–NIR (out of 2051) and XRF (out of 716) spectra.

Elements	UF		GA	
	vis–NIR	XRF	vis–NIR	XRF
As	890	369	604	331
Cd	838	334	610	201
Cu	1024	380	353	342
Pb	1364	354	558	316
Zn	567	311	337	301
Mn	943	193	557	18

**Table 4:** Statistics of the prediction model performance for soil PTEs concentration (mg/kg) using the fused spectra (validation data set).

Element	Data Set	R${}^{2}$	RMSE	Bias
	vis–NIR + XRF (Full-range)	0.76	60.9	17.1
As	vis–NIR + XRF (UF-selected)	0.69	66.9	17.8
	vis–NIR + XRF (GA-selected)	0.77	59.7	15.8
	vis–NIR + XRF (Full-range)	0.77	5.85	0.57
Cd	vis–NIR + XRF (UF-selected)	0.77	4.95	0.44
	vis–NIR + XRF (GA-selected)	0.77	4.04	0.44
	vis–NIR + XRF (Full-range)	0.75	10.85	-0.72
Cu	vis–NIR + XRF (UF-selected)	0.74	10.84	-2.16
	vis–NIR + XRF (GA-selected)	0.75	10.21	-0.40
	vis–NIR + XRF (Full-range)	0.85	401	43.0
Pb	vis–NIR + XRF (UF-selected)	0.86	389	42.2
	vis–NIR + XRF (GA-selected)	0.89	350	14.6
	vis–NIR + XRF (Full-range)	0.68	666	35.0
Zn	vis–NIR + XRF (UF-selected)	0.75	592	10.9
	vis–NIR + XRF (GA-selected)	0.71	659	-21.0
	vis–NIR + XRF (Full-range)	0.74	583	29.3
Mn	vis–NIR + XRF (UF-selected)	0.65	677	58.3
	vis–NIR + XRF (GA-selected)	0.76	563	13.3

## Data Availability

The field data and resulting data sets presented in this study are available on request from the corresponding author.
